# Outcomes following non-operative management for severe liver trauma: a UK multicentre observational study

**DOI:** 10.1186/s13049-026-01576-7

**Published:** 2026-03-06

**Authors:** Adam Brooks, Danielle Joyce, Santiago Gouveia, Marta Burak, Angelo LaValle, John-Joe Reilly, Alfred Adiamah, Thomas Diacon, Lauren Blackburn, Georgia Melia, Samuel Kitchen, Iver Anders Gaski, Christine Gaarder, Paal Aksel Næss, David N. Naumann

**Affiliations:** 1 East Midlands Major Trauma Centre, Queens Medical Centre, Nottingham, UK; 2 Department of Trauma and Emergency General Surgery, University Hospitals Birmingham NHS Foundation Trust, Birmingham, UK; 3 Department of Radiology, University Hospitals Birmingham NHS Foundation Trust, Birmingham, UK; 4 Department of Traumatology, Oslo University Hospital, Oslo, Norway; 5 University of Birmingham, Birmingham, UK; 6 Medical Technologies Innovation Facility (MTIF), Part of Nottingham Trent University, Nottingham, UK; 7 Institute of Inflammation and Ageing, University of Birmingham, Birmingham, UK

**Keywords:** Severe liver injury, Trauma, Non-operative management, High grade, Liver, Hepatobiliary injury

## Abstract

**Background:**

Patients with severe liver injury may carry a high risk of complications with significant mortality. Non-operative management (NOM) is increasingly common for severe injuries and may be associated with lower morbidity when compared with surgery.

**Objective:**

To evaluate the incidence of NOM for severe liver trauma (American Association for the Surgery of Trauma (AAST) Grade IV and V) and compare outcomes for NOM vs operative management.

**Methods:**

All patients admitted between 2012–2022 with severe liver trauma to Birmingham and Nottingham Major Trauma Centres (MTCs) were identified from a validated dataset. Outcomes were compared between those managed by surgery vs treated by NOM. Adjusted multivariable logistic regression models were used to determine the odds ratio (OR) and 95% confidence interval (95% CI) for surgical management, survival rates, and development of liver-specific complications (adjusting for age, sex, ISS, AAST grade, polytrauma).

**Results:**

There were 190 patients; median age 28 years (IQR 20–41); 134 (71%) were male. Median ISS was 27 (IQR 17–41). Overall mortality was 7% (14/190). 122/190 (64%) patients were managed initially by NOM, with only 8/122 (7%) requiring subsequent surgery. Multivariable logistic regression models showed higher ISS, lower SBP on admission, Grade V injuries and penetrating trauma to be independent predictors for surgical treatment. 34/190(18%) patients had liver-specific complications. There was no difference between NOM and operative management groups for 30-day mortality (*p* = 0.145), but patients in the NOM group had shorter ICU (*p* < 0.001) and total lengths of stay (*p* < 0.001) compared to the operative group.

**Conclusion:**

In this modern MTC setting, a high proportion of patients with severe liver trauma were managed by NOM with a low failure rate. Overall mortality rate was low, but liver-specific complications were common. These data support the evolution of traumatic liver injury management in the UK and favour NOM even in severe liver injuries where patient physiology allows.

**Trial registration:**

This study was approved by Nottingham University Hospital Clinical Audit Team (ID Reference 22-709C) and University Hospitals Birmingham NHS Foundation Trust Clinical Audit Registration and Management System (ID Reference CARMS 19146). Consent for participation in the study was not obtained as this was a large retrospective audit with anonymised data.

**Level of evidence:**

Level III.

## Background

The liver is one of the most commonly injured abdominal organs in major trauma and can be associated with a high risk of morbidity and mortality [1, 2]. Modern management of liver injuries has evolved towards increasing use of non-operative management (NOM), even for patients with high grade liver injuries [3, 4]. Important components include haemodynamic stability and the use of damage control resuscitation (DCR). A retrospective analysis of American Association of the Surgery of Trauma (AAST) IV and V injuries, that explored outcomes over intervening years before and after introduction of DCR principles, found an increased success rate of NOM, from 54% pre-DCR to 74% in the DCR group [5].

The tendency to favour NOM has been aided by some important advances in trauma care. The higher sensitivity and specificity of computed tomography (CT) scanning for liver injuries and improved resuscitation have facilitated the adoption of NOM in patients with liver injury. Nonetheless, the majority of currently available literature focuses on those patients presenting with isolated AAST Grade I and II liver injuries, which lend themselves with little controversy to NOM as they are most often haemodynamically stable.

The aim of the current study was to investigate the proportion of patients with severe liver trauma managed by NOM and the outcomes of these patients in a multicentre UK Major Trauma Centre (MTC) cohort, including the rate of failure of NOM. These data may help to better inform trauma practitioners about management strategies for these severe injuries and their prognosis, with a particular focus on NOM.

## Methods

### Study design and setting

A multi-centre collaborative observational study of patients with severe liver injury (defined as AAST grade 4 or 5 [6]) from 2012–2022 inclusive was undertaken at two high volume MTCs in the cities of Birmingham and Nottingham, UK. Institutional approval was granted at both sites prior to data collection. This study aimed to undertake an epidemiological descriptive study and a comparative study within the cohort of patients. Patients who were haemodynamically unstable were taken directly for surgery, but stable patients had a contrast enhanced Computed Tomography (CT) scan in order to inform their management. Included patients who had a CT scan initially were then either transferred to interventional radiology, theatre or a level 1 ward. At both centres included in this study, interventional radiology provides a 24 h on call service, but neither have hybrid CT scanners.

### Patient selection

Patients of all ages were eligible for inclusion if they met the criteria for AAST Grade 4 or 5 injury during the study period. Firstly, potentially eligible patients were identified using data from the Trauma Audit and Research Network (TARN), by searching for all patients with an Abbreviated Injury Score (AIS) for the abdomen of 3 or higher and cross-referencing this with contemporaneous records of patients with liver injuries in each Major Trauma Service. The Computed Tomography (CT) reports were then viewed for each potentially eligible patient by the study investigators for AAST scores assigned to liver injuries, and patients were included if a score of 4 or 5 was recorded. Where there was no score assigned, the images were reviewed by a Consultant Radiologist to assign scores according to AAST definitions. Patients who went straight to theatre without a CT scan were graded according to the intra-operative findings, and operative reports were reviewed to confirm AAST grade 4 or 5 liver injury.

### Data collection

Data were retrieved from a combination of sources including TARN records and local electronic medical records. Data included demographic details (age, sex), injury details (mechanism of injury, Injury Severity Score (ISS), individual AIS scores, AAST liver injury grade, Probability of Survival (Ps) calculated by TARN) and physiological data (heart rate (HR), systolic blood pressure (SBP) and Glasgow Coma Scale (GCS)) from the prehospital and Emergency Department (ED). Polytrauma was defined as more than one anatomical region scored for injury by TARN. Management strategies were recorded, including NOM, surgery and interventional radiology, and outcome data was collected. TARN calculates Ps by comparing patients with the same profile and attributes on the TARN database to determine the percentage of probability of survival.

### Outcomes

The outcomes of interest were failure of NOM (i.e. the patient still required surgery after a period of time of unsuccessful NOM), 30-day mortality, Glasgow Outcome Scale (GOS), liver-specific complications and length of stay (LOS). These covariates were chosen by author consensus decision. Hospital-free days and Intensive Care Unit (ICU)-free days were calculated as 30 minus the LOS in hospital or ICU respectively. Where there was death or longer LOS than 30 days this was a value of 0. NOM was defined as conservative management without surgical intervention (including patients who had interventional angiography and/or embolisation), and operative management was defined as abdominal surgery for the purpose of exploring and treating trauma to the liver. NOM included serial abdominal examinations, repeat liver function blood tests, monitoring of vital signs.

### Data analysis

Data are summarised using number and percentage for categorical data and median and interquartile range (IQR) for continuous data. Univariable comparisons were made using Fisher’s exact test for categorical data and Mann–Whitney U tests for continuous data. For all analyses, a *p* value < 0.05 from a two-tailed test was considered statistically significant. Univariable and multivariable logistic regression models were used to determine the odds ratio (OR) and 95% confidence intervals (95% CI) for surgical management, adjusted for age, sex, ISS, SBP in ED, AAST grade of liver injury and penetrating trauma. The odds ratio was also investigated using similar univariable and multivariable logistic regression models adjusted for age, sex, ISS, AAST Grade, polytrauma, surgery and the presence of liver complications. The variables were chosen a priori as factors thought likely to influence the outcome.

## Results

### Patient characteristics

There were 190 patients in the study cohort, with a median age of 28 (IQR 20–41); 134/190 (71%) were male. The median ISS was 27 (IQR 17–41). Patient characteristics are summarised in Table 1, with some comparison between patients initially managed by NOM and operative management (OM). Patients in the NOM group had a statistically significant higher probability of survival, lower prehospital HR, as well as better physiology in ED (higher SBP, higher GCS and lower HR). There was a higher proportion of patients with penetrating trauma in the surgery group (i.e. gunshot wounds (GSW) and stabbings).

**Table 1:** Patient characteristics for the study cohort, with univariable comparisons between those managed initially by NOM or OM

Characteristic	All (N-190)	NOM *n* = 122	OM *N* = 68	*p*-value
Age, median (IQR)	28 (IQR 20–41)	27 (19–41)	28 (20–41)	0.690
Male sex, n (%)	134 (71)			
ISS, median (IQR)	27 (17–41)	26 (17–36)	31 (17–43)	0.128
AAST Grade, n (%)				
* Grade IV*	148 (78)	100 (82)	48 (71)	0.100
* Grade V*	42 (22)	22 (18)	20 (29)	
Mechanism of injury, n (%)				
* Vehicle incident*	108 (57)	74 (61)	34 (50)	< 0.001*
* Stabbing*	34 (18)	10 (8)	24 (35)	
* Fall*	32 (17)	23 (19)	9 (13)	
* Blow*	10 (5)	10 (8)	0 (0)	
* Crush*	5 (3)	5 (4)	0 (0)	
* GSW*	1 (1)	0 (0)	1 (1)	
Polytrauma, n (%)†	146 (77)	91 (75)	55 (81)	0.324
Probability of survival (IQR)	98 (93–99)	99 (96–99)	97 (83–99)	0.008*
Prehospital physiology, median (IQR)				
* SBP, mmHg*	114 (96–132)	116 (99–132)	110 (86–131)	0.231
* HR, min*^*−1*^	104 (87–125)	100 (83–115)	118 (102–138)	< 0.001*
* GCS*	15 (12–15)	15 (14–15)	15 (11–15)	0.670
ED Physiology				
* SBP, mmHg*	117 (95–133)	121 (102–134)	106 (80–132)	0.022*
* HR, min*^*−1*^	103 (85–120)	98 (80–116)	112 (94–131)	< 0.001*
* GCS*	15 (13–15)	15 (14–15)	15 (4–15)	0.008*
Outcomes				
* 30 day mortality*	14 (7)	6 (5)	8 (12)	0.145
* GOS 5*	121 (64)	80 (66)	41 (60)	0.468
* Hospital-free days*	16 (0–24)	20 (1–25)	10 (0–21)	< 0.001*
* ICU-free days*	26 (14–29)	28 (22–30)	19 (6–26)	< 0.001*
* Liver-specific complications*	34 (18)	17 (14)	17 (25)	0.056

*IQR* interquartile range, *ISS* Injury Severity Score, *AAST* American Association for the Surgery of Trauma, *ED* Emergency Department, *SBP* systolic blood pressure, *HR* heart rate, *GCS* Glasgow Coma Scale, *OM* operative management, *NOM* non-operative management

^*^Statistically significant

^†^Polytrauma is defined as injuries in ≥ 2 organ systems

### Management strategies

**Fig. 1:**
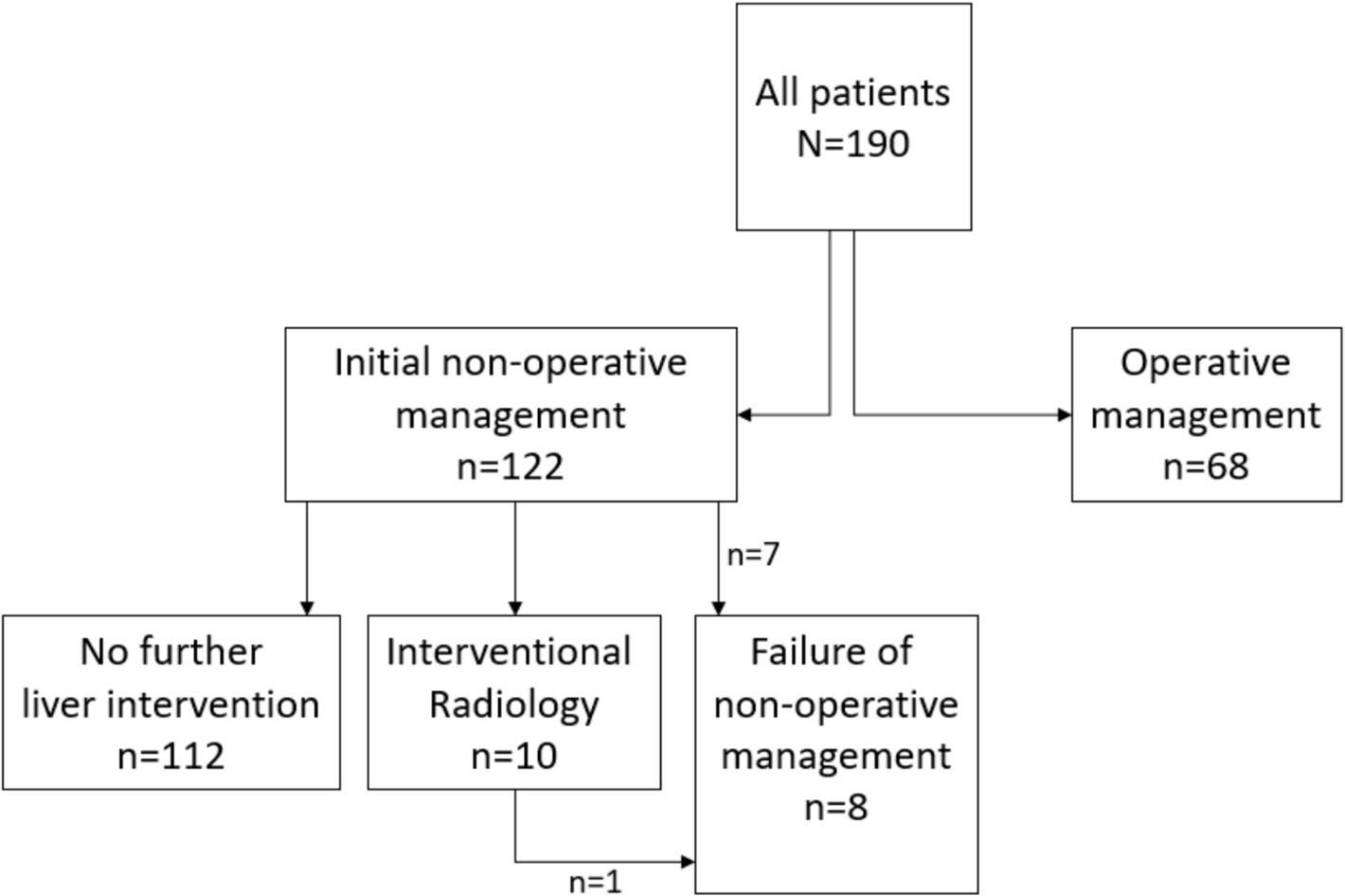
Flow diagram summarising the management strategies for the study cohort

Figure 1 summarises the management strategies for patients in the study cohort. There were 122/190 (64%) patients managed initially by NOM of whom 8 (7%) failed. There were 68 (46%) patients treated by operative management from the outset. Ten patients underwent angiography by an Interventional Radiologist, with 7 having embolisation and 3 having angiography only (no embolisation required). One of these patients required subsequent surgery. Table 2 summarises the univariable and multivariable logistic regression models of the likelihood of surgical management based on patient and injury characteristics. This model showed that patients with lower ISS, lower ED SBP, Grade 5 injuries and penetrating trauma were significantly more likely to have surgery in the multivariable model that also adjusted for age and sex (all* p* < 0.05). Table 3 summarises the characteristics of the patients managed initially by NOM, with comparison between those with successful and failed initial NOM. Although the median SBP appeared lower in the group that failed initial NOM for the prehospital (90 *vs* 116 mmHg) and ED (102 *vs* 122 mmHg) measurements, these did not reach statistical significance.

**Table 2:** Likelihood of surgical management of liver injuries according to (a) univariable and (b) multivariable logistic regression models

Characteristic	Univariable analysis		Multivariable analysis	
	OR (95% CI)	*p*-value	OR (95% CI)	*p*-value
Age	1.00 (0.98, 1.02)	0.989	1.00 (0.98, 1.02)	0.968
Male sex	1.12 (0.59, 2.19)	0.730	0.56 (0.23, 1.34)	0.194
ISS	1.02 (1.00, 1.04)	0.100	0.99 (0.98, 0.99)	< 0.001*
ED SBP	0.99 (0.98, 0.99)	0.020*	0.99 (0.98, 1.00)	0.035*
AAST grade 5	1.89 (0.94, 3.81)	0.0723	2.85 (1.18, 7.07)	0.021*
Penetrating	6.51 (2.97, 15.3)	< 0.001*	20.7 (7.22, 66.2)	< 0.001*

*OR* odds ratio, *95% CI* 95% confidence interval, *ISS* Injury Severity Score, *ED* Emergency Department, *AAST* American Association for the Surgery of Trauma, *SBP* systolic blood pressure

^*^Comparison for AAST grade 5 is all other patients included in the study

**Table 3:** Patient characteristics for the subgroup managed initially by NOM, with comparison between patients with successful and failed NOM

Characteristic	Successful NOM *n* = 114	Failed NOM *N* = 8	*p*-value
Age, median (IQR)	28 (20–42)	26 (13–35)	0.357
Male sex, n (%)	79 (69)	6 (75)	> 0.999
ISS, median (IQR)	25 (16–36)	30 (25–46)	0.182
AAST Grade, n (%)			0.634
* Grade IV*	94 (82)	6 (75)	
* Grade V*	20 (18)	2 (25)	
Prehospital physiology, median (IQR)			
* SBP, mmHg*	116 (102–133)	90 (69–122)	0.098
* HR, min*^*−1*^	100 (83–115)	100 (82–120)	0.996
* GCS*	15 (14–15)	15 (13–15)	0.963
ED Physiology			
* SBP, mmHg*	122 (107–135)	102 (80–124)	0.060
* HR, min*^*−1*^	98 (80–113)	111 (93–124)	0.148
* GCS*	15 (14–15)	15 (14–15)	0.637

*IQR* interquartile range, *ISS* Injury Severity Score, *AAST* American Association for the Surgery of Trauma, *ED* Emergency Department, *SBP* systolic blood pressure, *HR* heart rate, *GCS* Glasgow Coma Scale, *NOM* non-operative management

## Other outcomes

### Whole cohort

There were 14/190 (7%) patients who died within 30 days of injury (Table 1). The GOS was recorded as 5 (Good Recovery) for 121/176 (69%) patients. For the remainder of survivors there were 30/176 (17%) recorded as scoring 4 (Moderate Disability) and 5/176 (3%) with a score of 3 (Severe disability). There were 34/190 (18%) patients who had a liver-specific complication. These included bile leak/biloma (*n* = 20), abscess/infection (*n* = 7), death relating to severity of liver trauma (*n* = 2), persistent ductal dilatation (*n* = 2), necrosis (*n* = 1), thrombosis (*n* = 1) and post-traumatic cyst (*n* = 1). The median number of hospital-free and ICU-free days for the study cohort were 16 (IQR 0–24) and 26 (14–29) days respectively.

### According to non-operative management

When 30-day mortality was compared between the patients initially managed by NOM *vs* OM, the latter appeared higher but there was no statistically significant difference (6/122 (5%) *vs* 8/68 (12%) respectively; *p* = 0.145). Surgery was also not a significant factor in the likelihood of survival with good GOS when adjusted for age, sex, ISS, AAST grade, polytrauma and liver complications (Table 4). When patients managed by NOM were compared to those managed operatively, they had higher hospital-free days (20 (IQR 1–25) *vs* 10 (IQR 0–21) days respectively;* p* < 0.001) and higher ICU-free days (28 (IQR 22–30) *vs* 19 (IQR 6–26) days respectively; *p* < 0.001).

**Table 4:** Likelihood of survival with good recovery (Glasgow Outcome Scale 5) according to (a) univariable and (b) multivariable logistic regression models

**Characteristic**	(a) **Univariable analysis**		(b) **Multivariable analysis**	
	**OR (95% CI)**	***p*-value**	**OR (95% CI)**	***p*-value**
**Age**	0.99 (0.97, 1.01)	0.182	0.98 (0.95, 0.99)	0.040*
**Male sex**	1.03 (0.48, 2.14)	0.811	1.08 (0.44, 2.60)	0.862
**ISS**	0.92 (0.90, 0.95)	< 0.001*	0.92 (0.88, 0.95)	< 0.001*
**AAST grade 5**	0.89 (0.40, 2.12)	0.785	1.14 (0.44, 3.11)	0.793
**Polytrauma**	0.22 (0.05, 0.66)	0.017*	1.22 (0.24, 5.03)	0.794
**Surgery**	0.67 (0.33, 1.34)	0.251	0.90 (0.39, 2.13)	0.802
**Liver complication**	0.60 (0.27, 1.37)	0.208	0.47 (0.18, 1.24)	0.124

*OR* odds ratio, *95% CI* 95% confidence interval, *ISS* Injury Severity Score, *ED* Emergency Department, *AAST* American Association for the Surgery of Trauma, *SBP* systolic blood pressure

### Factors affecting outcomes

On univariable logistic regression, survival with a good GOS was less likely with higher ISS and for polytrauma patients (Table 4a) However, surgery vs NOM was not associated with survival with good GOS; only the ISS and age remained statistically significant on the multivariable model that adjusted for sex, ISS, AAST grade 5, polytrauma, surgery and liver complications (Table 4b).

## Discussion

The main finding from our study of patients with severe (AAST Grade 4 and 5) liver injury following trauma was that there was a 93% success rate of initial 64% NOM, and an overall mortality of 7%. Patients were predominately young, male, and mostly admitted following polytrauma. Within the study cohort, 18% of patients were found to have liver complications, with no significant difference between NOM and surgical management. As might be expected, our data confirm that surgical management was more likely for patients with higher AAST grade, lower SBP and those with penetrating trauma. Although length of stay outcomes were better for those managed with NOM than surgery, significant differences in overall mortality and GOS outcomes were not found when comparing the two groups of NOM vs surgery.

The demographics of this cohort were consistent with those reported across the literature with similar injuries [7, 8], with severe liver injuries tending to be associated with young males [9]. The management of those with complex liver injuries has changed over time, with the conservative approach of NOM becoming increasingly used [3, 10]. With the development and widely adopted use of CT in trauma management, NOM has become more favourable; this may be due to increased sensitivity of diagnostic trauma CT allowing for confidence in a conservative approach in managing liver injuries [3, 11].

Those who had surgery in this cohort were more likely to have penetrating trauma and worse physiology. However, when multivariable analysis adjusted for these factors, patients who had a laparotomy were not more likely to have poorer outcomes. Clinical decision making is a complex process and has been shown to be a combination of both intuitive and subconscious processes along with analytical and conscious processes [12]. Literature has demonstrated that surgeon factors, such as experience and training, confidence in a procedure and their skillset as well as previous similar patient cases and the need to balance risk [13], all have influence in the decision-making process [14, 15]. The current study could not determine which of these factors determined the decision-making processes for each patient, but the outcomes and low NOM failure rates suggest that in these high volume MTCs the decisions have been appropriately made on a case-by-case basis.

Although NOM is increasingly favoured for liver trauma, the majority of the literature has focused primarily on patients with isolated low grade liver injuries, which tend to lend themselves to NOM since they are most often haemodynamically stable [16]. However, in polytrauma patients and a cohort such as this, it is well documented that higher ISS is consistent with poorer outcomes in liver injury [17], perhaps reflecting the multisystem injuries sustained as a result of the trauma, rather than the severity of the liver injury in isolation [18, 19]. Our findings, of success of NOM in high grade liver injuries is consistent with the previously reported retrospective study that found success of NOM in over 70% of high grade AAST IV and V injuries [3]. The association between haemodynamic factors and operative intervention in our data is consistent with the currently available guidelines from the World Society of Emergency Surgery [20], which defines any grade of liver injury with haemodynamically instability as ‘severe’ and further suggests that such patients if non-responder to resuscitative measures should undergo operative intervention. This is consistent with a key finding of a number of studies [21–23] that have highlighted that prompt and individualised intervention, utilising NOM for haemodynamically stable patients, including those with contrast extravasation.

Rozycki et al. [24] described a cohort of patients with severe liver trauma. They described angioembolisation as its own category, whereas a large portion of the literature includes embolization as part of NOM. Rozycki et al. [24] reported a small number of patients who underwent embolization which was similar to our study and Gaski et al. [4], suggesting that the role of embolization in high grade liver trauma is limited although associated with good survival. The current study was not able to determine the risk of complications according to embolization or non-embolisation due to low numbers, but future studies should directly address this question.

This study provides evidence that NOM even in high grade liver injuries is safe in the context of haemodynamic stability. The AAST Grading of liver injury alone, whilst helpful in defining extent of liver damage and potentially useful in predicting sequalae, does not tend to be used in isolation in the clinical management decision making, which focuses on patient physiology and clinical presentation [25].

## Limitations

This study has several limitations, including those associated with its retrospective and observational design, such as risk of selection bias, the possibility of missing data and the non-randomisation of such data. Although a logistical regression model helped minimise confounding factors in the dataset, only associations (rather than causality) can be made between liver injuries and subsequent management. Since this study included patients with polytrauma in the UK with severe liver trauma, other injuries and their subsequent management were not addressed. Variables such as social determinants of health, comorbidities, use of anticoagulants, and continuous physiology would be helpful when considering the management of such patients, although these data are not captured by any available trauma registries. Greater details about specific injuries would be more helpful when managing these patients clinically, including the individual proportions of the AAST grading including haematoma, laceration and vascular injury, and specific CT findings, but were not included in the current study due to unavailability of data. Further analyses regarding the failure of NOM and the subsequent interventions, blood products use and damage control resuscitation protocol with a focus on the operative management would be useful to add to a comprehensive overview of the management of severe liver injuries, including a comparison between the liver related complications in both types of management. Multivariable models were limited in the number of variables that could be used due to the relatively low number of patients in the cohort. Further analyses with higher number of patients would be required to more fully explore all of the potentially significant variables of interest. As liver injuries were graded by two different centres, and grades are sometimes subjective, there may have been some potential differences in grading assessments between individuals. However, this was a pragmatic retrospective observational study and future prospective trials may wish to assure data using inter-observance consistency testing. Furthermore, with the limited sample size, although expected for this type of injury, the outcomes in this study may not be fully representative of the entire UK population.

## Conclusion

Haemodynamically stable patients with high AAST grade (4 and 5) liver injury can be managed successfully with NOM, with a low failure rate. Although there was a 1 in 5 rate of liver-related complications, overall mortality was low. These data support the evolution of traumatic liver injury management in the UK towards non-operative management even in severe liver injuries for stable patients.

## Data Availability

Data from the current study can be made available upon reasonable request to the corresponding author.

## Abbreviations

AAST: American Association for the Surgery of Trauma
AIS: Abbreviated Injury Score
CI: Confidence Interval
CT: Computed Tomography
DCR: Damage Control Resusictation
ED: Emergency Department
GCS: Glasgow Coma Sclae
GOS: Glasgow Outcome Scale
GSW: Gunshot Wound
HR: Heart Rate
ICU: Intensive Care Unit
IQR: Interquartile Range
ISS: Injury Severity Score
LOS: Length of Stay
MTC: Major Trauma Centre
NOM: Non-Operative Management
OR: Odds Ratio
Ps: Probability of Survival
SBP: Systolic Blood Pressure
TARN: Trauma Audit and Research Network
